# Supplementary material for: Socioeconomic Inequalities as a Cause of Health Inequities in Spain: A Scoping Review
Source: Healthcare (Basel). 2023 Nov 24;11(23):3035. doi: 10.3390/healthcare11233035 (PMC10706339; doi:10.3390/healthcare11233035)
Supplement: Supplementary file 1 [file healthcare-11-03035-s001.zip › healthcare-2689021-supplementary.pdf]

Supplementary Material

Table S1: Complete list of references included in the review.

1. Pascual-Sáez, M.; Cantarero-Prieto, D.; Lanza-León, P. The Dynamics of Health Poverty in Spain during the Economic Crisis (2008–2016). *Health Policy (New. York).* **2019**, *123*, 1011–1018, doi:10.1016/j.healthpol.2019.06.009.

2. Álvarez-Gálvez, J.; Rodero-Cosano, M.L.; Salinas-Pérez, J.A.; Gómez-Baya, D. Exploring the Complex Associations Among Social Determinants of Health in Andalusia After the 2008 Financial Crisis. *Soc. Indic. Res.* **2019**, *141*, 873–893, doi:10.1007/s11205-018-1845-z.

3. Bartoll, X.; Palència, L.; Malmusi, D.; Suhrcke, M.; Borrell, C. The Evolution of Mental Health in Spain during the Economic Crisis. *Eur. J. Public Health* **2014**, *24*, 415–418, doi:10.1093/eurpub/ckt208.

4. García-Goñi, M.; Nuño-Solinís, R.; Orueta, J.F.; Paolucci, F. Is Utilization of Health Services for HIV Patients Equal by Socioeconomic Status? Evidence from the Basque Country. *Int. J. Equity Health* **2015**, *14*, 1–10, doi:10.1186/s12939-015-0215-6.

5. Forcadell-Díez, L.; Gotsens, M.; Leon-Gomez, B.B.; Pérez, G. Social Inequalities in Fertility in Women Residing in Urban Neighbourhoods in Spain: A Multilevel Approach. *Matern. Child Health J.* **2020**, *24*, 267–274, doi:10.1007/s10995-020-02875-w.

6. Moreno-Maldonado, C.; Rivera, F.; Ramos, P.; Moreno, C. Measuring the Socioeconomic Position of Adolescents: A Proposal for a Composite Index. *Soc. Indic. Res.* **2018**, *136*, 517–538, doi:10.1007/s11205-017-1567-7.

7. Hurtado, J.L.; Bacigalupe, A.; Calvo, M.; Esnaola, S.; Mendizabal, N.; Portillo, I.; Idigoras, I.; Millán, E.; Arana-Arri, E. Social Inequalities in a Population Based Colorectal Cancer Screening Programme in the Basque Country. *BMC Public Health* **2015**, *15*, doi:10.1186/s12889-015-2370-5.

8. Ibáñez, B.; Galbete, A.; Goñi, M.J.; Forga, L.; Arnedo, L.; Aizpuru, F.; Librero, J.; Lecea, O.; Cambra, K. Socioeconomic Inequalities in Cardiometabolic Control in Patients with Type 2 Diabetes. *BMC Public Health* **2018**, *18*, 1–9, doi:10.1186/s12889-018-5269-0.

9. Barroso, C.; Abásolo, I.; Cáceres, J.J. Health Inequalities by Socioeconomic Characteristics in Spain: The Economic Crisis Effect. *Int. J. Equity Health* **2016**, *15*, 1–12, doi:10.1186/s12939-016-0346-4.

10. Capurro, D.A.; Davidsen, M. Socioeconomic Inequalities in Dental Health among Middle-Aged Adults and the Role of Behavioral and Psychosocial Factors: Evidence from the Spanish National Health Survey. *Int. J. Equity Health* **2017**, *16*, 1–9, doi:10.1186/s12939-017-0529-7.

11. Abásolo, I.; Saez, M.; López-Casasnovas, G. Financial Crisis and Income-Related Inequalities in the Universal Provision of a Public Service: The Case of Healthcare in Spain. *Int. J. Equity Health* **2017**, *16*, 1–14, doi:10.1186/s12939-017-0630-y.

12. Merino-Ventosa, M.; Urbanos-Garrido, R.M. Changes in Income-Related Inequalities in Cervical Cancer Screening during the Spanish Economic Crisis: A Decomposition Analysis. *Int. J. Equity Health* **2018**, *17*, 1–12, doi:10.1186/s12939-018-0894-x.

13. Pedrós Barnils, N.; Eurenius, E.; Gustafsson, P.E. Self-Rated Health Inequalities in the Intersection of Gender, Social Class and Regional Development in Spain: Exploring Contributions of Material and Psychosocial Factors. *Int. J. Equity Health* **2020**, *19*, 1–14, doi:10.1186/s12939-020-01202-7.

14. Arrospide, A.; Machón, M.; Ramos-Goñi, J.M.; Ibarrondo, O.; Mar, J. Inequalities in Health-Related Quality of Life According to Age, Gender, Educational Level, Social Class, Body Mass Index and Chronic Diseases Using the Spanish Value Set for Euroquol 5D-5L Questionnaire. *Health Qual. Life Outcomes* **2019**, *17*, 1–10, doi:10.1186/s12955-019-1134-9.

15. Rocha, K.B.; Perez, K.; Rodriguez-Sanz, M.; Muntaner, C.; Alonso, J.; Borrell, C. Inequalities in Mental Health in the Spanish Autonomous Communities: A Multilevel Study. *Span. J. Psychol.* **2015**, *18*, E27, doi:10.1017/sjp.2015.28.

16. Solé-Auró, A.; Martín, U.; Rodríguez, A.D. Educational Inequalities in Life and Healthy Life Expectancies among the 50-plus in Spain. *Int. J. Environ. Res. Public Health* **2020**, *17*, doi:10.3390/ijerph17103558.

17. López, J.C.; Castrillo, P.G.; González-Álvarez, M.A. Mortalidad y Estatus Socioeconómico En La España de Principios Del Siglo XXI. *Investig. Reg. - J. Reg. Res.* **2019**, *3*, 227–240.

18. Esteban y Peña, M.M.; Fernández Velasco, E.; Jiménez García, R.; Hernández Barrera, V.; Fernandez del Pozo, I. Salud e Incidencia y Diferencias En Vulnerabilidad Territorial de La Ciudad de Madrid. *Rev. Esp. Salud Publica* **2020**, *94*, 202004020, doi:10.4321/s1135-57272020000100012.

19. Orueta, J.F.; García-Álvarez, A.; Alonso-Morán, E.; Vallejo-Torres, L.; Nuño-Solinis, R. Socioeconomic Variation in the Burden of Chronic Conditions and Health Care Provision - Analyzing Administrative Individual Level Data from the Basque Country, Spain. *BMC Public Health* **2013**, *13*, doi:10.1186/1471-2458-13-870.

20. Orueta, J.F.; Nuño-Solinís, R.; García-Alvarez, A.; Alonso-Morán, E. Prevalence of Multimorbidity According to the Deprivation Level among the Elderly in the Basque Country. *BMC Public Health* **2013**, *13*, doi:10.1186/1471-2458-13-918.

21. Urbanos-Garrido, R.M. Social Inequalities in Health: Measuring the Contribution of Housing Deprivation and Social Interactions for Spain. *Int. J. Equity Health* **2012**, *11*, 1–14, doi:10.1186/1475-9276-11-77.

22. Terán, J.M.; Varea, C.; Juárez, S.; Bernis, C.; Bogin, B. Social Disparities in Low Birth Weight among Spanish Mothers during the Economic Crisis (2007-2015). *Nutr. Hosp.* **2018**, *35*, 129–141, doi:10.20960/NH.2095.

23. Zapata-Moya, Á.R.; Willems, B.; Bracke, P. The (Re)Production of Health Inequalities through the Process of Disseminating Preventive Innovations: The Dynamic Influence of Socioeconomic Status. *Heal. Sociol. Rev.* **2019**, *28*, 177–193, doi:10.1080/14461242.2019.1601027.

24. Bilal, U.; Hill-Briggs, F.; Sánchez-Perruca, L.; Del Cura-González, I.; Franco, M. Association of Neighbourhood Socioeconomic Status and Diabetes Burden Using Electronic Health Records in Madrid (Spain): The Heart Healthy Hoods Study. *BMJ Open* **2018**, *8*, 1–9, doi:10.1136/bmjopen-2017-021143.

25. Garrido-Cumbrera, M.; Borrell, C.; Palència, L.; Espelt, A.; Rodríguez-Sanz, M.; Pasarín, M.; Kunst, A. Social Class Inequalities in the Utilization of Health Care and Preventive Services in Spain, a Country with a National Health System. *Int. J. Heal. Serv.* **2010**, *40*, 525–542, doi:10.2190/HS.40.3.h.

26. Puig-Barrachina, V.; Malmusi, D.; Martínez, J.; Benach, J. Monitoring Social Determinants of Health Inequalities: The Impact of Unemployment among Vulnerable Groups. *Int. J. Heal. Serv.* **2011**, *41*, 459–482, doi:10.2190/HS.41.3.d.

27. Vives, A.; Vanroelen, C.; Amable, M.; Ferrer, M.; Moncada, S.; Llorens, C.; Muntaner, C.; Benavides, F.; Benach, J. Employment Precariousness in Spain: Prevalence, Social Distribution, and Population-Attributable Risk Percent of Poor Mental Health. *Int. J. Heal. Serv.* **2011**, *41*, 625–646, doi:10.2190/HS.41.4.b.

28. Belzunegui-Eraso, A.; Pastor-Gosálbez, I.; Puig-Andreu, X.; Valls-Fonayet, F. Risk of Exclusion in People with Disabilities in Spain: Determinants of Health and Poverty. *Int. J. Environ. Res. Public Health* **2018**, *15*, doi:10.3390/ijerph15102129.

29. Tamayofonseca, N.; Nolasco, A.; Moncho, J.; Barona, C.; Irles, M.Á.; Más, R.; Girón, M.; Gómezbeneyto, M.; Pereyrazamora, P. Contribution of the Economic Crisis to the Risk Increase of Poor Mental Health in a Region of Spain. *Int. J. Environ. Res. Public Health* **2018**, *15*, 1–16, doi:10.3390/ijerph15112517.

30. Saez, M.; López-Casasnovas, G. Assessing the Effects on Health Inequalities of Differential Exposure and Differential Susceptibility of Air Pollution and Environmental Noise in Barcelona, 2007–2014. *Int. J. Environ. Res. Public Health* **2019**, *16*, 4–6, doi:10.3390/ijerph16183470.

31. Marí-Dell'olmo, M.; Gotsens, M.; Pasarín, M.I.; Rodríguez-Sanz, M.; Artazcoz, L.; de Olalla, P.G.; Rius, C.; Borrell, C. Socioeconomic Inequalities in COVID-

19 in a European Urban Area: Two Waves, Two Patterns. *Int. J. Environ. Res. Public Health* **2021**, *18*, 1–12, doi:10.3390/ijerph18031256.

32. Rajmil, L.; López-Aguilà, S.; Penina, A.M.; Bustos, A.M.; Sanz, M.R.; Guiteras, P.B. Desigualdades Sociales En La Salud Mental Infantil En Cataluña. *An. Pediatr.* **2010**, *73*, 233–240, doi:10.1016/j.anpedi.2010.02.022.

33. Tornero Patricio, S.; Charris-Castro, L.; Granero Asencio, M.; Daponte Codina, A. Influence of Postcode on Paediatric Admissions in Seville. *An. Pediatr.* **2017**, *87*, 320–329, doi:10.1016/j.anpedi.2016.12.001.

34. Cebrecos, A.; Domínguez-Berjón, M.F.; Duque, I.; Franco, M.; Escobar, F. Geographic and Statistic Stability of Deprivation Aggregated Measures at Different Spatial Units in Health Research. *Appl. Geogr.* **2018**, *95*, 9–18, doi:10.1016/j.apgeog.2018.04.001.

35. Aguilar-Palacio, I.; Carrera-Lasfuentes, P.; Solsona, S.; Sartolo, M.T.; Rabanaque, M.J. Utilización de Servicios Sanitarios En Ancianos (España 2006-2012): Influencia Del Nivel de Salud y de La Clase Social. *Aten. Primaria* **2016**, *48*, 235–243, doi:10.1016/j.aprim.2015.01.016.

36. Compés Dea, M.L.; Olivan Bellido, E.; Feja Solana, C.; Aguilar Palacio, I.; García-Carpintero Romero Del Hombrebueno, G.; Adiego Sancho, B. Construcción de un índice de privación por zona básica de salud en Aragón a partir de datos de censo de 2011 [Construction of a deprivation index by Basic Healthcare Area in Aragon using Population and Housing Census 2011]. Revista *Española de Salud Pública* **2018**, *10*, e201812087.

37. Martín, U.; Malmusi, D.; Bacigalupe, A.; Esnaola, S. Migraciones Internas En Espana Durante El Siglo Xx: Un Nuevo Eje Para El Estudio de Las Desigualdades Sociales En Salud. *Gac. Sanit.* **2012**, *26*, 9–15, doi:10.1016/j.gaceta.2011.06.005.

38. Arias-de la Torre, J.; Artazcoz, L.; Molina, A.J.; Fernández-Villa, T.; Martín, V. Desigualdades En Salud Mental En Población Trabajadora de España: Un Estudio Basado En La Encuesta Nacional de Salud. *Gac. Sanit.* **2016**, *30*, 339–344, doi:10.1016/j.gaceta.2016.02.011.

39. Arias-de la Torre, J.; Molina, A.J.; Fernández-Villa, T.; Artazcoz, L.; Martín, V. Mental Health, Family Roles and Employment Status inside and Outside the Household in Spain. *Gac. Sanit.* **2019**, *33*, 235–241, doi:10.1016/j.gaceta.2017.11.005.

40. Bilal, U.; Glass, T.A.; del Cura-Gonzalez, I.; Sanchez-Perruca, L.; Celentano, D.D.; Franco, M. Neighborhood Social and Economic Change and Diabetes Incidence: The HeartHealthyHoods Study. *Heal. Place* **2019**, *58*, 102149, doi:10.1016/j.healthplace.2019.102149.

41. Crespo-Cebada, E.; Urbanos-Garrido, R.M. Equity and Equality in the Use of GP Services for Elderly People: The Spanish Case. *Health Policy (New. York).* **2012**, *104*, 193–199, doi:10.1016/j.healthpol.2011.10.007.

42. Crespo-Cebada, E.; Urbanos-Garrido, R.M. Equity and Equality in the Use of GP Services for Elderly People: The Spanish Case. *Health Policy (New. York).* **2012**, *104*, 193–199, doi:10.1016/j.healthpol.2011.10.007.

43. Cainzos-Achirica, M.; Capdevila, C.; Vela, E.; Cleries, M.; Bilal, U.; Garcia-Altes, A.; Enjuanes, C.; Garay, A.; Yun, S.; Farre, N.; et al. Individual Income, Mortality and Healthcare Resource Use in Patients with Chronic Heart Failure Living in a Universal Healthcare System: A Population-Based Study in Catalonia, Spain. *Int. J. Cardiol.* **2019**, *277*, 250–257, doi:10.1016/j.ijcard.2018.10.099.

44. Pérez-Hernández, B.; García-Esquinas, E.; Graciani, A.; Guallar-Castillón, P.; López-García, E.; León-Muñoz, L.M.; Banegas, J.R.; Rodríguez-Artalejo, F. Social Inequalities in Cardiovascular Risk Factors Among Older Adults in Spain: The Seniors-ENRICA Study. *Rev. Española Cardiol. (English Ed.* **2017**, *70*, 145–154, doi:10.1016/j.rec.2016.05.010.

45. Haeberer, M.; León-Gómez, I.; Pérez-Gómez, B.; Tellez-Plaza, M.; Rodríguez-Artalejo, F.; Galán, I. Social Inequalities in Cardiovascular Mortality in Spain from an Intersectional Perspective. *Rev. Española Cardiol. (English Ed.* **2020**, *73*, 282–289, doi:10.1016/j.rec.2019.07.022.

46. Rueda, S. Health Inequalities among Older Adults in Spain: The Importance of Gender, the Socioeconomic Development of the Region of Residence, and Social Support. *Women's Heal. Issues* **2012**, *22*, e483–e490, doi:10.1016/j.whi.2012.07.001.

47. Bilal, U.; Cainzos-Achirica, M.; Cleries, M.; Santaeugènia, S.; Corbella, X.; Comin-Colet, J.; Vela, E. Socioeconomic Status, Life Expectancy and Mortality in a Universal Healthcare Setting: An Individual-Level Analysis of >6 Million Catalan Residents. *Prev. Med. (Baltim).* **2019**, *123*, 91–94, doi:10.1016/j.ypmed.2019.03.005.

48. De Bont, J.; Diáz, Y.; Casas, M.; Garciá-Gil, M.; Vrijheid, M.; Duarte-Salles, T. Time Trends and Sociodemographic Factors Associated with Overweight and Obesity in Children and Adolescents in Spain. *JAMA Netw. Open* **2020**, *3*, 1–13, doi:10.1001/jamanetworkopen.2020.1171.

49. Duarte-Salles, T.; Pasarín, M.I.; Borrell, C.; Rodríguez-Sanz, M.; Rajmil, L.; Ferrer, M.; Pellise, F.; Balague, F. Social Inequalities in Health among Adolescents in a Large Southern European City. *J. Epidemiol. Community Health* **2011**, *65*, 166–173, doi:10.1136/jech.2009.090100.

50. Maynou, L.; Saez, M.; Lopez-Casasnovas, G. Has the Economic Crisis Widened the Intraurban Socioeconomic Inequalities in Mortality? The Case of Barcelona,

Spain. *J. Epidemiol. Community Health* **2014**, *70*, 114–124, doi:10.1136/jech-2013-203447.

51. Fernández, S.C.; Ajuria, A.F.; Martín, J.J.; Murphy, M.J. The Impact of the Economic Crisis on Unmet Dental Care Needs in Spain. *J. Epidemiol. Community Health* **2015**, *69*, 880–885, doi:10.1136/jech-2014-204493.

52. Hernández-Yumar, A.; Wemrell, M.; Alessón, I.A.; López-Valcárcel, B.G.; Leckie, G.; Merlo, J. Socioeconomic Differences in Body Mass Index in Spain: An Intersectional Multilevel Analysis of Individual Heterogeneity and Discriminatory Accuracy. *PLoS One* **2018**, *13*, 1–23, doi:10.1371/journal.pone.0208624.

53. Morteruel, M.; Rodriguez-Alvarez, E.; Martin, U.; Bacigalupe, A. Inequalities in Health Services Usage in a National Health System Scheme: The Case of a Southern Social European Region. *Nurs. Res.* **2018**, *67*, 26–34, doi:10.1097/NNR.0000000000000256.

54. Barriuso-Lapres, L.; Hernando-Arizaleta, L.; Rajmil, L. Social Inequalities in Mental Health and Health-Related Quality of Life in Children in Spain. *Pediatrics* **2012**, *130*, doi:10.1542/peds.2011-3594.

55. Zoni, A.C.; Domínguez-Berjón, M.F.; Esteban-Vasallo, M.D.; Velázquez-Buendía, L.M.; Blaya-Nováková, V.; Regidor, E. Socioeconomic Inequalities in Injuries Treated in Primary Care in Madrid, Spain. *J. Public Heal. (United Kingdom)* **2017**, *39*, 45–51, doi:10.1093/pubmed/fdw005.

56. Amengual-Moreno, M.; Calafat-Caules, M.; Carot, A.; Correia, A.R.R.; Río-Bergé, C.; Plujà, J.R.; Pascual, C.V.; Ventura-Gabarró, C. Social Determinants of the Incidence of Covid-19 in Barcelona: A Preliminary Ecological Study Using Public Data. *Rev. Esp. Salud Publica* **2020**, *94*.

57. Larrañaga, I.; Santa-Marina, L.; Begiristain, H.; Machón, M.; Vrijheid, M.; Casas, M.; Tardón, A.; Fernández-Somoano, A.; Llop, S.; Rodriguez-Bernal, C.L.; et al. Socio-Economic Inequalities in Health, Habits and Self-Care during Pregnancy in Spain. *Matern. Child Health J.* **2013**, *17*, 1315–1324, doi:10.1007/s10995-012-1134-4.

58. Nuñez, O.; Barranco, M.R.; Fernández-Navarro, P.; Sanchez, D.R.; Fernández, M.Á.L.; Santamaría, M.P.; Sánchez, M.-J. Deprivation Gap in Colorectal Cancer Survival Attributable to Stage at Diagnosis: A Population-Based Study in Spain. *Cancer Epidemiol.* **2020**, *68*, doi:https://doi.org/10.1016/j.canep.2020.101794.
